# Supplementary material for: Exploring Diversity and Polymer Degrading Potential of Epiphytic Bacteria Isolated from Marine Macroalgae
Source: Microorganisms. 2022 Dec 19;10(12):2513. doi: 10.3390/microorganisms10122513 (PMC9786321; doi:10.3390/microorganisms10122513)
Supplement: Supplementary file 1 [file microorganisms-10-02513-s001.zip › Supplementary tables.pdf]

**Supplementary table S1: CFU counts and total number of bacterial isolates preserved from intertidal macroalgal samples on different media**

| SAMPLES (ID) | CFU/g of sample (No. of isolates preserved) | | | | | | Total |
|---|---|---|---|---|---|---|---|
| | TSBA | TSBA (1:100) | R2A | MA | VNSS | SWC | |
| ***Ulva sp.*, Malwan (GA-MAL)** | $4.9 \times 10^7$ (12) | NG* | $4.0 \times 10^5$ (6) | $9.1 \times 10^7$ (2) | $1.2 \times 10^7$ (11) | $1.1 \times 10^7$ (11) | **42** |
| ***Sargassum polycystum*, Malwan (MBA)** | $6.0 \times 10^6$ (7) | $4.0 \times 10^6$ (2) | $6.0 \times 10^6$ (4) | $2.0 \times 10^7$ (3) | $3 \times 10^6$ (2) | $9.1 \times 10^9$ (6) | **24** |
| ***Pterocladia musciformis* Malwan (MRA)** | $6.1 \times 10^7$ (13) | $4.0 \times 10^3$ (7) | $5.0 \times 10^5$ (11) | $1.4 \times 10^8$ (4) | $1.6 \times 10^8$ (6) | $1.4 \times 10^8$ (6) | **47** |
| ***Sargassum polycystum*, Anjuna (LLAB)** | $3.9 \times 10^8$ (5) | $2.2 \times 10^3$ (16) | $2.03 \times 10^5$ 17) | $2.8 \times 10^8$ (4) | $1.9 \times 10^8$ (3) | $1.7 \times 10^8$ (7) | **52** |
| ***Dictyota sp.*, Anjuna (SAB)** | $9.6 \times 10^5$ (5) | $1.46 \times 10^4$ (25) | $1.9 \times 10^5$ (38) | $1.9 \times 10^8$ (3) | $1.2 \times 10^8$ (4) | $1.9 \times 10^8$ (6) | **81** |
| ***Padina antillarum*, Cabo-De-Rama (CDR-SL)** | $1.4 \times 10^8$ (15) | $1.7 \times 10^8$ (5) | $2.0 \times 10^4$ (8) | $4.7 \times 10^8$ (19) | $3.6 \times 10^8$ (14) | $4.8 \times 10^7$ (13) | **74** |
| ***Sargassum polycystum*, Kunkeshwar (LLKUN)** | $1.1 \times 10^8$ (10) | NG* | $2.0 \times 10^6$ (7) | $1.1 \times 10^9$ (3) | $1.1 \times 10^9$ (11) | $1.3 \times 10^9$ (9) | **40** |
| **Total isolates** | **67** | **55** | **91** | **38** | **51** | **58** | **=360** |

**Supplementary table S2: List of total identified strains and their polymer degrading potentials (sample wise) with NCBI accession number.**

**1. Cellulose, 2. Xylan, 3. Pectin, 4. Starch**

| Sr. No. | Strain | Taxonomy | 1 | 2 | 3 | 4 |
|---|---|---|---|---|---|---|
| **SAB** | | **TSBA** | | | | |
| 1 | SAB 1 (MW173255) | *Bacillus coreaensis* (97.9) | - | + | + | - |
| 2 | SAB 2 (MT734468) | *Oceanobacillus caeni* (100) | + | - | + | - |
| 3 | SAB 3 (MW173256) | *Vibrio alginolyticus* (99.7) | - | + | + | + |
| | | **TSBAD** | | | | |
| 4 | SAB 1 (MW180946) | *Vibrio fortis* (100) | - | - | - | - |
| 5 | SAB 2 (MW173258) | *Klebsiella oxytoca* (100) | - | - | - | - |
| 6 | SAB 3 (MH707108) | *Kosakonia sacchari* (99.9) | - | - | - | - |
| 7 | SAB 5 (MH707109) | *Enterobacter sichuanensis* (99.5) | - | - | - | - |
| 8 | SAB 7 (OP379650) | *Cellulosimicrobium aquatile* (99.5) | + | + | + | ++ |

| | | | | | | |
|---|---|---|---|---|---|---|
| 9 | SAB 8 (MH707111) | *Cellulosimicrobium funkei* (94.8) | - | - | - | ++ |
| 10 | SAB 10 (MW180945) | *Streptomyces albidoflavus* (99.8) | + | - | + | +++ |
| 11 | SAB 14 (MH707116) | *Gordonia bronchialis* (95.36) | - | - | - | - |
| 12 | SAB 16 (MT947150) | *Brachybacterium squillarum* (100) | - | - | - | ++ |
| 13 | SAB 17 (MW173259) | *Bacillus infantis* (99.8) | + | + | + | + |
| 14 | SAB 18 (MH707117) | *Bacillus infantis* (99.7) | + | + | + | + |
| 15 | SAB 19 (MH707118) | *Fictibacillus enclensis* (99.7) | + | - | + | - |
| 16 | SAB 20 (MH707119) | *Streptomyces hyaluromycini* (99.8) | + | - | + | - |
| 17 | SAB 23 (MH707120) | *Brachybacterium squillarum* (99.8) | - | - | - | - |
| 18 | SAB 24 (MH707121) | *Halobacillus yeomjeoni* (91.4) | - | - | - | - |
| 19 | SAB 25 (MH701858) | *Fredinandcohnia onubensis* (98.8) | + | - | - | - |
| 20 | SAB 26 (MH707122) | *Enterobacter asburiae* (99.6) | - | - | - | - |
| 21 | SAB 27 (MW173260) | *Bacillus acidiceler* (99.8) | - | - | - | - |
| | | **MA** | | | | |
| 22 | SAB 1 (MH707126) | *Vibrio fortis* (99.8) | - | + | - | + |
| 23 | SAB 2 (MW173261) | *Lederbergia wuyishanensis (98.4)* | + | + | + | - |
| 24 | SAB 3 (LN999519) | *Marinomonas epiphytica* (100) | - | - | - | - |
| | | **R2A** | | | | |
| 25 | SAB 1 (MH707128) | *Enterobacter soli* (99.1) | + | + | - | - |
| 26 | SAB 2 (MH707129) | *Aeromonas bivalvium* (99.8) | + | + | - | + |
| 27 | SAB 3 (MH707130) | *Klebsiella michiganensis* (99.9) | - | + | - | - |
| 28 | SAB 4 (MH707131) | *Cytobacillus firmus* (99.4) | + | + | - | - |
| 29 | SAB 11 (MH707135) | *Gordonia bronchialis* (98.8) | - | + | - | - |
| 30 | SAB 12 (MH707136) | *Kocuria palustris* (99.5) | - | + | - | + |
| 31 | SAB 13 (MH707137) | *Isoptericola chiayiensis* (99.7) | - | + | - | ++ |
| 32 | SAB 14 (MH707138) | *Roseomonas mucosa* (100) | + | + | - | + |
| 33 | SAB 15 (MH707139) | *Gordonia westfalica* (99.9) | + | - | - | - |
| 34 | SAB 16 (MW173262) | *Bacillus pseudomycoides* (100) | + | - | - | + |
| 35 | SAB 18 (MH707140) | *Pantoea anthophila* (100) | - | - | - | + |
| 36 | SAB 19 (MH707141) | *Salinimonas lutimaris* (99.4) | - | - | - | - |
| 37 | SAB 20 (MH707142) | *Neobacillus cucumis* (98.5) | + | + | + | + |

| | | | | | | |
|---|---|---|---|---|---|---|
| 38 | SAB 21 (MH707143) | *Bacillus altitudinis* (100) | - | - | - | - |
| 39 | SAB 23 (MH707144) | *Priestia filamentosus* (100) | + | - | - | + |
| 40 | SAB 25 (MW173263) | *Alkalihalobacillus algicola* (100) | - | + | - | - |
| 41 | SAB 27 (MH707146) | *Priestia aryabhattai* (99.9) | + | + | - | + |
| 42 | SAB 28 (MH707147) | *Psychrobacter nivimaris* (100) | + | + | - | + |
| 43 | SAB 29 (MH707148) | *Kocuria palustris* (100) | - | + | - | - |
| 44 | SAB 30 (MH707149) | *Psychrobacter nivimaris* (100) | + | + | - | + |
| 45 | SAB 31 (MH707150) | *Enterobacter asburiae* (99.6) | - | - | - | - |
| 46 | SAB 32 (MT734469) | *Microbacetrium schleiferi* (99.17) | - | - | - | + |
| 47 | SAB 33 (MH707151) | *Staphylococcus warneri* (100) | - | - | - | - |
| 48 | SAB 34 (MH707152) | *Alkalihaloacillus algicola* (99.3) | + | + | - | + |
| 49 | SAB 35 (MH707153) | *Priestia filamentosa (*99.9) | + | - | - | - |
| 50 | SAB 36 (MH707154) | *Bacillus Altitudinis* (99.9) | - | - | - | - |
| 51 | SAB 37 (MH707155) | *Cobetia marina* (99.9) | - | + | - | - |
| 52 | SAB 38 (LN879432) | *Domibacillus epiphytica* (100) | - | - | - | - |
| 53 | SAB 39 (MH707156) | *Gordonia rubripertincta* (99.8) | - | - | + | + |
| 54 | SAB 40 (MH707157) | *Janibacter indicus* (99.6) | - | - | + | + |
| 55 | SAB 41 (MH707158) | *Vibrio barsiliensis* (99) | + | + | + | + |
| 56 | SAB 42 (MH707159) | *Bacillus aerophilus* (100) | - | + | - | - |
| 57 | SAB 45 (MH707160) | *Alkalihalobacillus algicola* (99.7) | + | + | + | + |
| 58 | SAB 52 (MH707161) | *Staphylococcus warneri* (100) | - | - | - | - |
| **SWC** | | | | | | |
| 59 | SAB1 (MH707162) | *Vibrio natriegens* (99.6) | + | + | + | ++ |
| 60 | SAB2 (MH707163) | *Pseudoalteromonas shioyasakiensis* (99.7) | - | + | + | + |
| 61 | SAB4 (MH707165) | *Vibrio neocaldonicus* (99.8) | + | + | + | +++ |
| 62 | SAB5 (MH707166) | *Rossellomorea marisflavi* (99.8) | - | + | + | + |
| 63 | SAB13 (MH707167) | *Pseudoalteromonas distincta* (99.9) | - | + | + | + |
| **VNSS** | | | | | | |
| 64 | SAB-1 (MH707168) | *Bacillus aerophilus* (96.4) | + | - | - | - |
| 65 | SAB-3 (MH707169) | *Vibrio neocaldonicus* (99.6) | - | - | + | + |
| 66 | SAB-10 (MH707170) | *Pseudoalteromonas tetraodonis* (97.9) | - | - | - | + |

| | | | | | | |
|---|---|---|---|---|---|---|
| 67 | SAB-11 (MH707171) | *Pseudoalteromonas agarivorans* (96.7) | - | - | + | + |
| **CDR-SL** | | **TSBA** | | | | |
| 68 | CDR-SL 1 (MH707181) | *Staphylocccus argenteus* (99.4) | - | - | - | - |
| 69 | CDR-SL 3 (MH707182) | *Vibrio owensii* (99.7) | + | - | + | + |
| 70 | CDR-SL 4 (MH707183) | *Staphylocccus argenteus* (100) | - | - | - | - |
| 71 | CDR-SL 5 (MH707184) | *Psychrobacter nivimaris* (99.9) | - | - | - | - |
| 72 | CDR-SL 7 (MH707186) | *Metabacillus halosaccharovorans* (99.4) | + | + | + | - |
| 73 | CDR-SL 8 (OP379651) | *Staphylocccus argenteus* (99.92) | - | + | - | + |
| 74 | CDR-SL 10 (MH701859) | *Vibrio variabilisi* (96.8) | - | - | + | - |
| 75 | CDR-SL 11 (MH707187) | *Vibrio neocaldonicus* (99.5) | + | - | + | - |
| 76 | CDR-SL 12 (MH707188) | *Staphylocccus argenteus* (100) | - | + | - | ++ |
| 77 | CDR-SL 14 (MH707189) | *Staphylocccus argenteus* (99.7) | - | + | - | ++ |
| 78 | CDR-SL 16 (MW174850) | *Micrococcus luteus* (99.5) | + | - | + | + |
| 79 | CDR-SL 20 (MH707190) | *Staphylococcus epidermidis* (100) | - | - | - | - |
| 80 | CDR-SL 21 (MH707191) | *Staphylococcus warneri* (100) | - | - | - | - |
| 81 | CDR-SL 22 (MH707192) | *Staphylococcus caprae* (100) | - | - | - | - |
| | | **TSBAD** | | | | |
| 82 | CDR-SL 1 (MH707193) | *Gordonia terrae* (99.7) | + | - | + | - |
| 83 | CDR-SL 2 (MH707194) | *Niallia circulans* (99.5) | + | - | + | - |
| 84 | CDR-SL 4 (MH707195) | *Micrococcus aloeverae* (99.9) | + | - | + | - |
| 85 | CDR-SL 5 (MH707196) | *Staphylococcus epidermidis* (99.8) | - | - | + | + |
| 86 | CDR-SL 7 (MH707197) | *Niallia circulans* (99.5) | - | + | + | + |
| | | **MA** | | | | |
| 87 | CDR-SL 1 (MH707198) | *Vibrio fortis* (99.9) | - | + | - | + |
| 88 | CDR-SL 3 (MH707199) | *Kocuria sediminis* (100) | + | + | + | ++ |
| 89 | CDR-SL 5 (MH707200) | *Kocuria palustris* (99.9) | + | + | + | + |
| 90 | CDR-SL 7 (MH707201) | *Planococcus okeanokoites* (99.9) | + | + | + | + |
| 91 | CDR-SL 8 (MH707202) | *Alteromonas macleodii* (99.6) | - | - | + | + |
| 92 | CDR-SL 10 (MH707203) | *Rossellomorea marisflavi* (99.8) | - | + | - | + |
| 93 | CDR-SL 11 (MH707204) | *Kocuria palustris* (99.6) | + | + | + | + |
| 94 | CDR-SL 12 (MH707205) | *Micrococcus terreus* (100) | - | + | + | + |

| | | | | | | |
|---|---|---|---|---|---|---|
| 95 | CDR-SL 13 (MH707206) | *Micrococcus endophyticus* (99.9) | - | + | + | + |
| 96 | CDR-SL 14 (MW174849) | *Kocuria palustris* (100) | + | - | + | + |
| 97 | CDR-SL 15 (LN879436) | *Luteimonas padinae* (100) | - | + | - | - |
| 98 | CDR-SL 16 (MH707207) | *Kocuria palustris* (100) | - | + | + | - |
| 99 | CDR-SL 18 (MH707208) | *Micrococcus endophyticus* (99.2) | + | + | + | + |
| 100 | CDR-SL 26 (MW180944) | *Streptomyces olivicoloratus* (100) | + | + | + | + |
| 101 | CDR-SL 27 (MT734471) | *Kocuria sediminis* (100) | - | - | + | [+] |
| 102 | CDR-SL 29 (MH707210) | *Brevundimonas vesicularis* (99) | - | - | + | + |
| 103 | CDR-SL 30 (MH707211) | *Kocuria palustris* (100) | - | - | + | + |
| 104 | CDR-SL 31 (MW180943) | *Paracoccus marcusii* (99.9) | - | - | + | + |
| 105 | CDR-SL 32 (MH707212) | *Planomicrobium okeanokoites* (99.8) | + | + | + | + |
| **R2A** | | | | | | |
| 106 | CDR-SL 6 (MH707215) | *Sphingomonas molluscorum* (99.8) | + | + | - | + |
| 107 | CDR-SL 8 (MH707217) | *Staphylococcus epidermidis* (99.7) | - | + | - | - |
| **SWC** | | | | | | |
| 108 | CDRSL1 (MW173264) | *Vibrio owensii* (99) | + | + | - | + |
| 109 | CDRSL2 (MH707218) | *Vibrio sonorensis* (99) | + | - | - | + |
| 110 | CDRSL6 (MH707219) | *Alteromonas macleodii* (99.6) | - | + | | + |
| 111 | CDRSL-7 SWC (MH707220) | *Vibrio hepatarius* (99.7) | + | + | + | + |
| 112 | CDRSL-8 (MH707221) | *Alteromonas macleodii* (99.7) | + | + | + | + |
| 113 | CDRSL-9 SWC (MH707222) | *Vibrio tubiashii* (99.1) | + | + | - | + |
| 114 | CDRSL-10 SWC (MH707223) | *Alteromonas macleodii* (99.6) | + | + | - | + |
| 115 | CDRSL-12 (MH707224) | *Alteromonas macleodii* (99.9) | + | + | - | + |
| 116 | CDRSL-16 (MH707225) | *Alteromonas macleodii* (99.1) | + | + | + | + |
| 117 | CDRSL-17 (MH707226) | *Alteromonas macleodii* (99.6) | + | ++ | + | ++ |
| 118 | CDRSL-18 (MH707227) | *Alteromonas marina* (99.6) | - | + | + | + |
| 119 | CDRSL-20 (MH707228) | *Bacillus altitudinis* (99.9) | + | + | + | + |
| 120 | CDRSL-23 (MH707229) | *Alteromonas macleodii* (99.9) | - | + | - | + |
| **VNSS** | | | | | | |
| 121 | CDR-SL-1 (MH707230) | *Vibrio corallilyticus* (97.8) | + | + | - | + |

| | | | | | | |
|---|---|---|---|---|---|---|
| 122 | CDR-SL-2 (MT947141) | *Alteromonas macleodii* (99.8) | + | + | - | + |
| 123 | CDR-SL-3 (MW173265) | *Alteromonas macleodii* (99.8) | - | - | - | + |
| 124 | CDR-SL-5 (MT947143) | *Vibrio maritimus* (99.0) | + | + | - | + |
| 125 | CDR-SL-6 (MH707233) | *Vibrio corallilyticus* (99.5) | + | + | - | + |
| 126 | CDR-SL-8 (MH707235) | *Alteromonas macleodii* (100) | - | + | - | + |
| 127 | CDR-SL-9 (MH707236) | *Vibrio coralliilyticus* (98.9) | + | + | - | + |
| 128 | CDR-SL-13 (MH707237) | *Vibrio tubiashii* (99.5) | + | - | - | + |
| 129 | CDR-SL-19 (MH707238) | *Brevundimonas vesicularis* (99.6) | - | + | - | + |
| 130 | CDR-SL-21 (MH707239) | *Photobacterium rosenbergii* (99.3) | - | - | - | - |
| 131 | CDR-SL-24 (MH707240) | *Vibrio tubiashii* (99.7) | + | + | - | + |
| 132 | CDR-SL-25 (MH707241) | *Vibrio tubiashii* (99.4) | + | + | + | + |
| **GAMAL** | | | **TSBA** | | | |
| 133 | GAMAL13 (MT634725) | *Micrococcus aloeverae* (100) | + | - | + | + |
| 134 | GAMAL10 (MT634723) | *Vibrio shilonii* (99.7) | + | + | - | +* |
| 135 | GAMAL11 (MT634724) | *Vibrio alginolyticus* (100) | + | + | + | + |
| | | | **MA** | | | |
| 136 | GAMAL7 (MT634736) | *Rossellomorea vietnamensis* (99.8) | + | - | + | + |
| 137 | GAMAL8 (MT734473) | *Pseudoalteromonas shioyasakiensis* (100) | + | + | +* | + |
| | | | **R2A** | | | |
| 138 | GAMAL1 (*MT734472*) | *Staphylococcus epidermidis (100)* | + | + | - | + |
| 139 | GAMAL13 (MW173267) | *Staphylococcus epidermidis (100)* | + | + | - | + |
| 140 | GAMAL11 (MW174844) | *Sanguibacter inulinus* (99.7) | - | - | - | + |
| 141 | GAMAL 2, R2A (MT634726) | *Exiguobacterium mexicanum* (100) | | + | | + |
| 142 | GAMAL3 R2A (MW173268) | *Rosellomorea oryzaecorticis* (96.7) | + | + | +* | - |
| 143 | GAMAL 12, R2A (MW174845) | *Exiguobacterium mexicanum* (100) | | + | | + |
| | | | **SWC** | | | |
| 144 | GAMAL1 (MT634727) | *Vibrio neocaledonicus* (100) | + | + | + | + |
| 145 | GAMAL2 (MT634728) | *Vibrio owensii* (100) | + | + | + | + |
| 146 | GAMAL3 (MT634729) | *Pseudoalteromonas undina* (99.8) | + | + | +* | + |
| 147 | GAMAL6 (MT634730) | *Shewanella fidelis* (100) | - | - | +* | - |
| 148 | GAMAL8 (OP379653) | *Bacillus infantis* (99.3%) | + | + | +* | + |

| | | | | | | |
|---|---|---|---|---|---|---|
| 149 | GAMAL9 (MT634731) | *Shewanella fidelis* (100) | + | + | +* | + |
| 150 | GAMAL10 (MT634732) | *Rosellomorea marisflavi* (99.9) | + | + | + | + |
| | | | **VNSS** | | | |
| 151 | GAMAL2 (MW173270) | *Pseudoalteromonas issachenkonii* (99.8) | + | + | - | + |
| 152 | GAMAL3 (MH703640) | *Sphingomonas desiccabilis* (99.3) | + | - | - | - |
| 153 | GAMAL8 (MW173272) | *Shewanella fidelis* (100) | + | + | - | +* |
| 154 | GAMAL 9, VNSS(MH703642) | *Streptomyces lavenduligriseus* (100) | + | + | + | - |
| 155 | GAMAL10 (MW174848) | *Staphylococcus epidermidis* (100) | + | - | - | - |
| 156 | GAMAL11 (MW173273) | *Bacillus altitudinis* (100) | - | + | - | - |
| 157 | GAMAL12 (MW174847) | *Bacillus altitudinis* (100) | + | + | - | - |
| 158 | GAMAL13 (MW174846) | *Shewanella fidelis* (100) | + | + | + | - |
| 159 | GAMAL14 (MW173271) | *Pseudoalteromonas undina (99.1)* | + | - | - | + |
| | | **MBA** | **TSBA** | | | |
| 160 | MBA2 (MT734492) | *Kocuria indica* (100) | + | + | ++ | +* |
| 161 | MBA5 (MT947147) | *Bacillus coreansis* (99.5) | + | - | + | + |
| 162 | MBA7 (MH701874) | *Bacillus megaterium* (99.8) | + | + | ++ | + |
| 163 | MBA3 (MT734493) | *Brevibacillus nitrificans* (100) | + | + | + | - |
| 164 | MBA4 (MW174838) | *Kocuria palustris* (100) | + | + | + | + |
| | | | **MA** | | | |
| 165 | MBA3 (MT734491) | *Moraxella osolensis* (99.3) | - | - | + | + |
| 166 | MBA2 (MT734490) | *Priestia megaterium* (99.9) | + | + | + | + |
| | | | **R2A** | | | |
| 167 | MBA1 (MT734486) | *Bacillus pseudomycoides* (99.4) | + | + | ++ | ++ |
| 168 | MBA3 (MT734487) | *Cellulomonas fimi* (99.5) | + | + | ++ | ++ |
| 169 | MBA4 (MT734488) | *Bacillus pseudomycoides* (98.8) | - | + | ++ | ++ |
| 170 | MBA5 (MT734489) | *Microbacterium hydrothermale* (99.9) | + | - | - | - |
| | | | **VNSS** | | | |
| 171 | MBA1 (MT947138) | *Staphylococcus epidermidis (100)* | + | - | + | - |
| 172 | MBA2 (MT947137) | *Staphylococcus epidermidis (100)* | + | + | + | + |
| 173 | MBA7 (MW173274) | *Pseudoalteromonas tetraodonis* (99.90) | + | + | + | + |
| | | **MRA** | **MA** | | | |
| 174 | MRA1 (MT734505) | *Vibrio nereis* (99.8) | + | - | + | + |

| | | | | | | |
|---|---|---|---|---|---|---|
| 175 | MRA2 (MT734506) | *Vibrio shilonii* (100) | + | + | - | +* |
| 176 | MRA4 (MT734507) | *Vibrio neocaledonicus* (100) | + | + | +* | +* |
| **R2A** | | | | | | |
| 177 | MRA1 (MT734494) | *Priestia megaterium* (100) | - | + | - | + |
| 178 | MRA2 (MT734495) | *Neobacillus drentensis* (99.7) | + | + | ++ | ++ |
| 179 | MRA3 (MW174833) | *Bacillus pseudomycoides* (98.9) | + | + | ++ | ++ |
| 180 | MRA7 (MW173276) | *Bacillus cereus* (99.9) | + | + | ++ | +* |
| 181 | MRA12 (MT735153) | *Bacillus medetierraneensis* (97.76) | + | + | + | + |
| 182 | MRA14 (MT734496) | *Priestia aryabhattai* (100) | + | - | +* | ++ |
| 183 | MRA15 (MT734497) | *Staphylococcus epidermidis* (100) | + | + | - | + |
| **MRA** | | | **SWC** | | | |
| 184 | MRA1 (MW173277) | *Microbacterium arborescens* (100) | + | + | + | - |
| 185 | MRA2 (MW173278) | *Pseudoalteromonas tetraodonis* (99.8) | + | + | + | - |
| 186 | MRA6 (MW173280) | *Vibrio jasicida* (100) | + | + | + | - |
| 187 | MRA8 (MW173281) | *Vibrio parahaemolyticus* (99.9) | + | + | + | +* |
| 188 | MRA10 (MW173279) | *Pseudoalteromonas tetraodonis* (99.9) | + | - | + | + |
| **VNSS** | | | | | | |
| 189 | MRA1 (MT947139) | *Shewanella fidelis* (100) | + | + | + | - |
| 190 | MRA2 (MW174836) | *Pseudoalteromonas undina* (100) | + | - | ++ | + |
| 191 | MRA5 (MT947140) | *Shewanella fidelis* (100) | + | + | - | - |
| 192 | MRA6 (MW173283) | *Vibrio azureus* (99.8) | + | + | ++ | - |
| 193 | MRA7 (MW174835) | *Pseudoalteromonas undina* (99.6) | + | + | ++ | - |
| **LLKUN** | | | **TSBA** | | | |
| 194 | LLKUN1 (MT734509) | *Vibrio neocaledonicus*, (100) | - | - | ++ | - |
| 195 | LLKUN2 (MT734510) | *Bacillus pseudomycoides* (98.62) | + | + | ++ | + |
| 196 | LLKUN4 (OP379652) | *Priestia aryabhattai* (99.9) | + | + | +* | + |
| 197 | LLKUN5 (MT734512) | *Microbacterium arborescens* (100) | + | - | - | - |
| 198 | LLKUN7 (MT734513) | *Vibrio neocaledonicus*, (100) | + | - | + | - |
| 199 | LLKUN9 (MT734514) | *Bacillus pseudomycoides* (98.6) | - | + | + | - |
| 200 | LLKUN14 (MW174839) | *Bacillus australimaris* (100) | + | - | +* | + |
| **MA** | | | | | | |

| | | | | | | |
|---|---|---|---|---|---|---|
| 201 | LLKUN4 (MT734508) | *Pseudoalteromonas undina* (99.8) | + | + | + | + |
| **R2A** | | | | | | |
| 202 | LLKUN2 (MT734515) | *Bacillus cereus* (100) | + | - | + | + |
| 203 | LLKUN3 (MT947152) | *Staphylococcus epidermidis* (100) | + | + | +* | - |
| 204 | LLKUN14 (MT734516) | *Microbacterium enclense* (100) | + | + | - | - |
| **SWC** | | | | | | |
| 205 | LLKUN4 (MW173284) | *Vibrio furnissi* (98.72) | - | - | - | - |
| 206 | LLKUN5 (MW174840) | *Pseudoalteromonas issachenkonii* (99.9) | + | + | + | - |
| 207 | LLKUN8 (MW173285) | *Bacillus cerus* (100) | + | - | +* | + |
| 208 | LLKUN9 (MW180941) | *Bacillus pseudomycoides* (100) | - | + | + | + |
| 209 | LLKUN10 (MW173286) | *Brevibacillus gelatini* (99.7) | + | - | + | - |
| 210 | LLKUN12 (MW173287) | *Alteromonas australica* (99.7) | + | + | - | - |
| 211 | LLKUN14 (MW173288) | *Microbacterium enclense* (100) | + | - | - | - |
| **VNSS** | | | | | | |
| 212 | LLKUN2 (MT734517) | *Pseudoalteromonas issachenkonii* (99.8) | + | + | ++ | + |
| 213 | LLKUN3 (MT734518) | *Vibrio hyugaensis* (100) | + | - | ++ | + |
| 214 | LLKUN5 (MT947136) | *Alteromonas fortis* (99.7) | + | + | ++ | + |
| 215 | LLKUN7 (MW173292) | *Alteromonas macleodii* (99.5) | + | + | - | + |
| 216 | LLKUN8 (MT947144) | *Bacillus canaveralius* (98.5) | + | - | - | + |
| 217 | LLKUN10 (MW173289) | *Pseudoalteromonas tetraodonis* (100) | + | + | ++ | + |
| 218 | LLKUN12 (MT947135) | *Staphylococcus epidermidis* (99.4) | + | + | ++ | + |
| **LLAB** | | **TSBA (1:100)** | | | | |
| 219 | LLAB2 (MT734482) | *Neobacillus drentensis* (99.1) | + | + | +* | + |
| **MA** | | | | | | |
| 220 | LLAB2 (MT734480) | *Vibrio neocaledonicus* (100) | + | - | + | + |
| 221 | LLAB7 (MT734481) | *Catenococcus thiocycli* (99.8) | + | + | + | + |
| 222 | LLAB8 (MW174843) | *Vibrio kanaloae* (100) | + | + | + | + |
| **R2A** | | | | | | |
| 223 | LLAB29 (MW174842) | *Micrococcus yunnanensis* (100) | + | - | - | + |
| 224 | LLAB28 (MT734485) | *Vibrio plantisponsor* (99.7) | - | + | - | - |
| 225 | LLAB26 (MT734484) | *Bacillus tequilensis* (100) | + | +* | - | + |
| 226 | LLAB22 (MW180940) | *Paenibacillus lacus* (99.2) | - | +* | - | - |

| | | | | | | |
|---|---|---|---|---|---|---|
| 227 | LLAB19 (MW174841) | *Brevibacillus limnophilus* (100) | - | - | - | - |
| 228 | LLAB18 (MT734483) | *Streptomyces albidoflavus* (99.8) | + | +* | - | + |
| 229 | LLAB15 (MT947146) | *Bacillus cereus* (99.4) | + | +* | - | + |
| | | **SWC** | | | | |
| 230 | LLAB1 (MT734474) | *Vibrio hyugaensis* (100) | + | - | - | - |
| 231 | LLAB2 (MT734475) | *Vibrio hyugaensis* (100) | + | + | + | - |
| 232 | LLAB3 (MT734476) | *Vibrio hyugaensis* (100) | + | - | - | + |
| 233 | LLAB5 (MT734477) | *Vibrio sonorensis* (100) | - | + | + | - |
| 234 | LLAB7 (MT734478) | *Pseudoalteromonas issachenkonii* (99.6) | + | + | - | +* |
| 235 | LLAB8 (MT734479) | *Vibrio hyugaensis* (100) | - | + | - | - |
| 236 | LLAB9 (MW173295) | *Vibrio owensii* (99.82) | + | + | + | +* |
| | | **VNSS** | | | | |
| 237 | LLAB4 (MW173296) | *Vibrio japonicas* (99.8) | + | - | +* | + |
| 238 | LLAB8 (OK161099) | *Vibrio alginolyticus* (100) | + | + | +* | + |

'+*' weakly positive

**Supplementary table S3: Sample wise distribution profile of unique and shared taxa**

| Genus | SAB | CDRSL | GAMAL | LLKUN | LLAB | MRA | MBA |
|---|---|---|---|---|---|---|---|
| ***Actinobacteria*** | | | | | | | |
| *Brachybacterium* | 2 | 0 | 0 | 0 | 0 | 0 | 0 |
| *Cellulomonas* | 0 | 0 | 0 | 0 | 0 | 0 | 1 |
| *Cellulosimicrobium* | 2 | 0 | 0 | 0 | 0 | 0 | 0 |
| *Gordonia* | 4 | 1 | 0 | 0 | 0 | 0 | 0 |
| *Isoptericola* | 1 | 0 | 0 | 0 | 0 | 0 | 0 |
| *Janibacter* | 1 | 0 | 0 | 0 | 0 | 0 | 0 |
| *Kocuria* | 2 | 7 | 0 | 0 | 0 | 0 | 2 |
| *Microbacterium* | 1 | 0 | 0 | 3 | 0 | 1 | 1 |
| *Micrococcus* | 0 | 5 | 1 | 0 | 1 | 0 | 0 |
| *Sanguibacter* | 0 | 0 | 1 | 0 | 0 | 0 | 0 |
| *Streptomyces* | 2 | 1 | 1 | 0 | 1 | 0 | 0 |
| ***Alphaproteobacteria*** | | | | | | | |
| *Brevundimonas* | 0 | 2 | 0 | 0 | 0 | 0 | 0 |
| *Luteimonas* | 0 | 1 | 0 | 0 | 0 | 0 | 0 |
| *Paracoccus* | 0 | 1 | 0 | 0 | 0 | 0 | 0 |
| *Roseomonas* | 1 | 0 | 0 | 0 | 0 | 0 | 0 |
| *Sphingomonas* | 0 | 1 | 1 | 0 | 0 | 0 | 0 |

| | | | | | | | |
|---|---|---|---|---|---|---|---|
| ***Bacilli*** | | | | | | | |
| *Alkalihalobacillus* | 3 | 0 | 0 | 0 | 0 | 0 | 0 |
| *Bacillus* | 9 | 1 | 4 | 8 | 2 | 3 | 4 |
| *Brevibacillus* | 1 | 0 | 0 | 1 | 1 | 0 | 1 |
| *Cytobacillus* | 1 | 0 | 0 | 0 | 0 | 0 | 0 |
| *Domibacillus* | 1 | 0 | 0 | 0 | 0 | 0 | 0 |
| *Exiguobacterium* | 0 | 0 | 2 | 0 | 0 | 0 | 0 |
| *Fictibacillus* | 1 | 0 | 0 | 0 | 0 | 0 | 0 |
| *Fredinandcohnia* | 1 | 0 | 0 | 0 | 0 | 0 | 0 |
| *Halobacillus* | 1 | 0 | 0 | 0 | 0 | 0 | 0 |
| *Lederbergia* | 1 | 0 | 0 | 0 | 0 | 0 | 0 |
| *Metabacillus* | 0 | 1 | 0 | 0 | 0 | 0 | 0 |
| *Niallia* | 0 | 2 | 0 | 0 | 0 | 0 | 0 |
| *Neobacillus* | 1 | 0 | 0 | 0 | 1 | 1 | 0 |
| *Oceanobacillus* | 1 | 0 | 0 | 0 | 0 | 0 | 0 |
| *Paenibacillus* | 0 | 0 | 0 | 0 | 1 | 0 | 0 |
| *Planococcus* | 0 | 1 | 0 | 0 | 0 | 0 | 0 |
| *Planomicrobium* | 0 | 1 | 0 | 0 | 0 | 0 | 0 |
| *Priestia* | 3 | 0 | 0 | 1 | 0 | 2 | 1 |
| *Rossellomorea* | 0 | 1 | 2 | 0 | 0 | 0 | 0 |
| *Staphylococcus* | 2 | 10 | 3 | 2 | 0 | 1 | 0 |
| ***Gammaproteobacteria*** | | | | | | | |
| *Aeromonas* | 1 | 0 | 0 | 0 | 0 | 0 | 0 |
| *Alteromonas* | 0 | 12 | 0 | 3 | 0 | 0 | 0 |
| *Catenococcus* | 0 | 0 | 0 | 0 | 1 | 0 | 0 |
| *Cobetia* | 1 | 0 | 0 | 0 | 0 | 0 | 0 |
| *Enterobacter* | 4 | 0 | 0 | 0 | 0 | 0 | 0 |
| *Klebsiella* | 2 | 0 | 0 | 0 | 0 | 0 | 0 |
| *kosakonia* | 1 | 0 | 0 | 0 | 0 | 0 | 0 |
| *Marinomonas* | 1 | 0 | 0 | 0 | 0 | 0 | 0 |
| *Moraxella* | 0 | 0 | 0 | 0 | 0 | 0 | 1 |
| *Pantoea* | 1 | 0 | 0 | 0 | 0 | 0 | 0 |
| *Photobacterium* | 0 | 1 | 0 | 0 | 0 | 0 | 0 |
| *Pseudoalteromonas* | 4 | 0 | 4 | 4 | 1 | 4 | 1 |
| *Psychrobacter* | 2 | 1 | 0 | 0 | 0 | 0 | 0 |
| *Salinimonas* | 1 | 0 | 0 | 0 | 0 | 0 | 0 |
| *Shewanella* | 0 | 0 | 4 | 0 | 0 | 2 | 0 |
| *Vibrio* | 7 | 15 | 4 | 4 | 11 | 6 | 2 |

Unique among SAB
Unique among CDRSL
Unique among LLAB
Unique among MBA
Unique among GAMAL
Unique among LLKUN

Shared among CDRSL/LLKUN
Shared among SAB/LLKUN/LLAB/MBA
Shared among SAB/CDRSL
Shared among SAB/CDRSL/MBA
Shared among SAB/MRA/MBA
Shared among SAB/LLKUN/MRA/MBA
Shared among CDRSL/GAMAL/LLAB
Shared among GAMAL/MRA
Shared among CDRSL/GAMAL
Shared among SAB/CDRSL/GAMAL/MRA
Shared among SAB/CDRSL/GAMAL/LLAB
Shared among SAB/GAMAL/LLKUN/LLAB/MRA/MBA
Shared among all

**Supplementary Table S4: Comparative analysis of the present study with previous reports of epiphytic bacterial community**

| S. NO | | Climate | Sampling sites | Algae | | Media | Taxa Identified (Genus) | Polymer activity |
|---|---|---|---|---|---|---|---|---|
| *1* | Present study | Tropical, Humid | Goa, India | *Rhodophyta* | *Pterocladia musciformis* | Soyabean casein digest (TSBA), Soyabean casein digest diluted (TSBAD), Marine agar (MA), reasoner's 2 agar (R2A), Seawater complex agar medium (SWC medium), Vaatanen nine salt solution agar medium (VNSSA medium) | *Vibrio, Bacillus, Pseudoalteromonas, Alteromonas, Staphylococcus, Kocuria, Micrococcus, streptomyces, Microbacterium, Shewanella, Marinomonas, Sphingomonas, Gordonia, Psychrobacter,* and *Enterobacter etc. (only abundant taxa for rare <1% refer to figure 3)* | Cellulase, Amylase, Xylanase, Pectinase, Sugarcane bagasse hydrolase |
| | | | | *Phaeophyta* | *Sargassum polycystum, Padina antillarum, and Dictyota sp.* | | | |
| | | | | *Chlorophyta* | *Ulva sp.* | | | |
| *2* | Barbato et al., 2022 [61] | Temperate | South portugal | *Rhodophyta* | *Asparagopsis taxiformis* | Marine agar, DSMZ medium 950 | *Alteromonas, Celeribacter, Photobacterium, Pseudoalteromonas, Shewanella, Vibrio, Bacillus, Fictibacillus, Halobacillus, Thalassobacillus, Lacinutrix, Lutibacter, Winogradskiella, Aestuariibacter, Alcanivorax, Micrococcus, Algibacter, Arenibacter, Cellulophaga, Zobellia, Terribacillus, Huaishuia, Labrenzia, Paracoccus,* and *Cobetia* | Agarase, Algnate, Pectinase, and Amylase |
| | | | | | *Sphaerococcus coronopifolius* | | | |
| | | | | *Pheophyta* | *Halopteris scoparia* | | | |

| | | | | | | | | |
|---|---|---|---|---|---|---|---|---|
| *3* | Abdullah et al., 2020 [15] | Tropical, Humid | Kedah, Malaysia | *Rhodophyta* | *Gracilaria sp.* | Marine agar, Actinomycetes isolation agar, Seawater agar, Seawater+ KBr, Seawater+ KI, Seawater+ NaCl, Seawater+$NH_4Cl$, and Seawater+$NaNO_3$ | *Bacillus, Labrenzia, Microbulbifer, Virgibacillus, Alkanivorax, Stapia, Flavobacterium, Solibacillus, Sphingobacterium, Streptomyces,* and *Erythrobacter* | NA |
| *4* | Naik et al., 2018 [17] | Tropical, Humid | Goa, India | *Chlorophyta* | *Ulva lactuca* | Seawater agar medium | *Vibrio, Bacillus,* and *Pseudomonas* | Cellulase, Amylase, protease, Agarase, Carrageenase |
| *5* | Hinojosa et al., 2018 [16] | Temprate | King George Island, Peninsula | *Rhodophyta* | *Pantoneura plocamioides, Plocamium cartilagineum* | Marine agar, Seawater agar | *Pseudoalteromonas, Paraglaciecola, Colwellia, Cellulophaga, Olleya, Lacinutrix,* and *Winogradskyella* | Agarase |
| | | | | *Phaeophyta* | *Himantothallus grandifolius* | | | |
| *6* | Wang et al., 2017 [60] | Sub-tropical | Nanhuangcheng Island | *Pheophyta* | *Laminaria japonica* | Seawater | *Paenibacillus, Bacillus, Leclercia, Isoptericola, Planomicrobium, Pseudomonas, Lysinibacillus,* and *Sphingomonas* | Alginate |
| | | | | | *Sargassum horneri* | | | |
| | | | | | *Sargassum siliquatrum* | | | |
| *7* | Alvarado et al., 2017 [53] | Temperate | King George Island, Peninsula | *Rhodophyta* | *Iridaea cordata, Pantoneura plocamioides* | Marine agar and Seawater agar | *Algibacter, Cellulophaga, Zobellia, Arthrobacter, Brachybacterium, Citrococcus, Labedella, Microbacterium, Micrococcus, Sanguibacter,* and *Salinibacterium* | Agarase |
| | | | | *Phaeophyta* | *Adenocystis utricularis* | | | |
| | | | | *Chlorophyta* | *Monostroma hariotii* | | | |
| *8* | Rodrigues et al 2017 [18] | Humid temperate oceanic | Wales, UK | *Chlorophyta* | *Ulva sp.* | Marine agar | *Vibrio, Halomonas, Cobetia, Psychromonas, Pseudoalteromonas, Zymobacter, Moritella, Photobacterium, Winogradskyella,* and *Allivibrio* | Ulvan, hydrolase, Carbohydrate sulphatase, and Cellulase |
| *9* | Vieira et al., 2016 [59] | Semi-tropical | Noumea lagoon, New Caledonia | *Pheophyta* | *Lobophora* | Marine agar (MA) | *Rugeria, Thalassomonas, Vibrio, Microbulbifer, Tanacibaculum, Paramoritella, Shimia, Erythrobacter, Muricauda,* and *Bacillus* | NA |

| | | | | | | | | |
|---|---|---|---|---|---|---|---|---|
| *10* | Albakosh et al., 2016 [9] | Subtropical temperate | Rooi Els, Cape town (South Africa) | *Phaeophyta* | *Splachinidium rugossum* | Thiosulphate citrate bile sucrose agar, Marine agar, Nutrient agar, Nutrient seawater agar | *Vibrio, Pseudoalteromonas, Alteromonas, Psychromonas, Pseudomonas, Neptumonas, Cobetia, Shewanella, Sphingomonas,* and *Sulfitobacter* | NA |
| *11* | Menzes et al., 2009 [52] | Tropical, Subtropical | Sao Sebastiao region and Sao Paulo State, Brazil | *Pheophyta* | *Sargassum sp.* | Glucose peptone yeast extract (GPY medium), Marine agar (MA), Tryptic soya agar (TSA) | *Arthrobacter, Bacillus, Brevundimonas, Dokdonia, Knoellia, Kocuria, Micrococcus, Nocardiodes, Ruegeria, Staphylococcus,* and *Vibrio* | NA |

## Supplementary Table S5: List of potential novel isolates.

| Sr. | Sample ID | Accession No. | 16S rRNA gene sequencing (% similarity) | Putative taxonomic affiliation | Remarks |
|---|---|---|---|---|---|
| 1 | SAB-1 TSBA | MW173255 | *Bacillus coreaensis* (97.9) | *Firmicutes* | |
| 2 | SAB-8 TSBAD | MH707111 | *Cellulosimicrobium funkei* (94.8) | *Actinobacteria* | |
| 3 | SAB-38 R2A | LN879432 | *Domibacillus epiphyticus* (100) | *Firmicutes* | *Domibacillus epiphyticus* sp. Nov. Verma et al., (2017) [76] |
| 4 | SAB-14 TSBAD | MH707116 | *Gordonia bronchialis* (95.3) | *Actinobacteria* | |
| 5 | SAB-24 TSBAD | MH707121 | *Halobacillus yeomjeoni (91.4)* | *Firmicutes* | |
| 6 | SAB-2 MA | MW173261 | *Lederbergia wuyishanensis* (98.4) | *Firmicutes* | |
| 7 | SAB-3 MA | LN999519 | *Marinomonas epiphytica* (100) | *Gamma-proteobacteria* | *Marinomonas epiphytica* sp. nov. Ojha et al., (2017) [37] |
| 8 | SAB-20 R2A | MH707142 | *Neobacillus Cucumis* (98.5) | *Firmicutes* | |
| 9 | SAB-1 VNSS | MH707168 | *Bacillus aerophilus* (96.4) | *Firmicutes* | |
| 10 | SAB-10 VNSS | MH707170 | *Pseudoalteromonas tetraodonis* (97.9) | *Gamma-proteobacteria* | |
| 11 | SAB-11 VNSS | MH707171 | *Pseudoalteromonas agarivornas* (96.7) | *Gamma-proteobacteria* | |
| 12 | CDRSL-10 TSBA | MH701859 | *Vibrio variabilis* (96.8) | *Gamma-proteobacteria* | |
| 13 | CDRSL-15 MA | LN879436 | *Luteimonas padinae* (100) | *Gamma-proteobacteria* | *Luteimonas padinae* sp. Nov. Verma et al., 2016 [77] |

| | | | | | |
|---|---|---|---|---|---|
| 14 | CDRSL-1 VNSS | MH707230 | *Vibrio corallilyticus* (97.8) | *Gamma-proteobacteria* | |
| 15 | GAMAL-3 R2A | MW173268 | *Rossellomorea oryzaecorticis* (96.7) | *Firmicutes* | |
| 16 | MRA-12 R2A | MT735153 | *Bacillus massilioalgeriensis* (97.13) | *Firmicutes* | |
| 17 | LLKUN-2 TSBA | MT734510 | *Bacillus pseudomycoides* (98.6) | *Firmicutes* | |
| 18 | LLKUN-9 TSBA | MT734514 | *Bacillus pseudomycoides* (98.6) | *Firmicutes* | |
| 19 | LLKUN-4 SWC | MW173284 | *Vibrio furnissi* (98.72) | *Gamma-proteobacteria* | |
| 20 | LLKUN-8 VNSS | MT947144 | *Bacillus canaveralius* (98.5) | *Firmicutes* | |

**Supplementary table S6: Raw sugarcane bagasse degradation potential of strains from macroalgae.**

| **Sr.** | **Isolates** | **Identity** | **Sugarcane bagasse** |
|---|---|---|---|
| 1 | GAMAL-2 SWC | *Vibrio owensii* | ++ |
| 2 | GAMAL-8 SWC | *Bacillus infantis* | ++ |
| 3 | GAMAL-10 SWC | *Rossellomorea marisflavi* | ++ |
| 4 | LLAB-2 TSBAD | *Neobacillus derentis* | ++ |
| 5 | LLKUN-4 TSBA | *Bacillus Pseudomycoides* | + |
| 6 | MRA-3 R2A | *Bacillus pseudomycoides* | + |
| 7 | MRA-7 R2A | *Bacillus cereus* | + |
| 8 | SAB 20 R2A | *Neobacillus cucumis* | W+ |
| 9 | SAB 18 TSBAD | *Bacillus infentis* | W+ |

***"++: highly positive; "+": positive; "W+": weakly positive**
