# Supplementary figures and images for: Exploring Diversity and Polymer Degrading Potential of Epiphytic Bacteria Isolated from Marine Macroalgae
Source: Microorganisms. 2022 Dec 19;10(12):2513. doi: 10.3390/microorganisms10122513 (PMC9786321; doi:10.3390/microorganisms10122513)

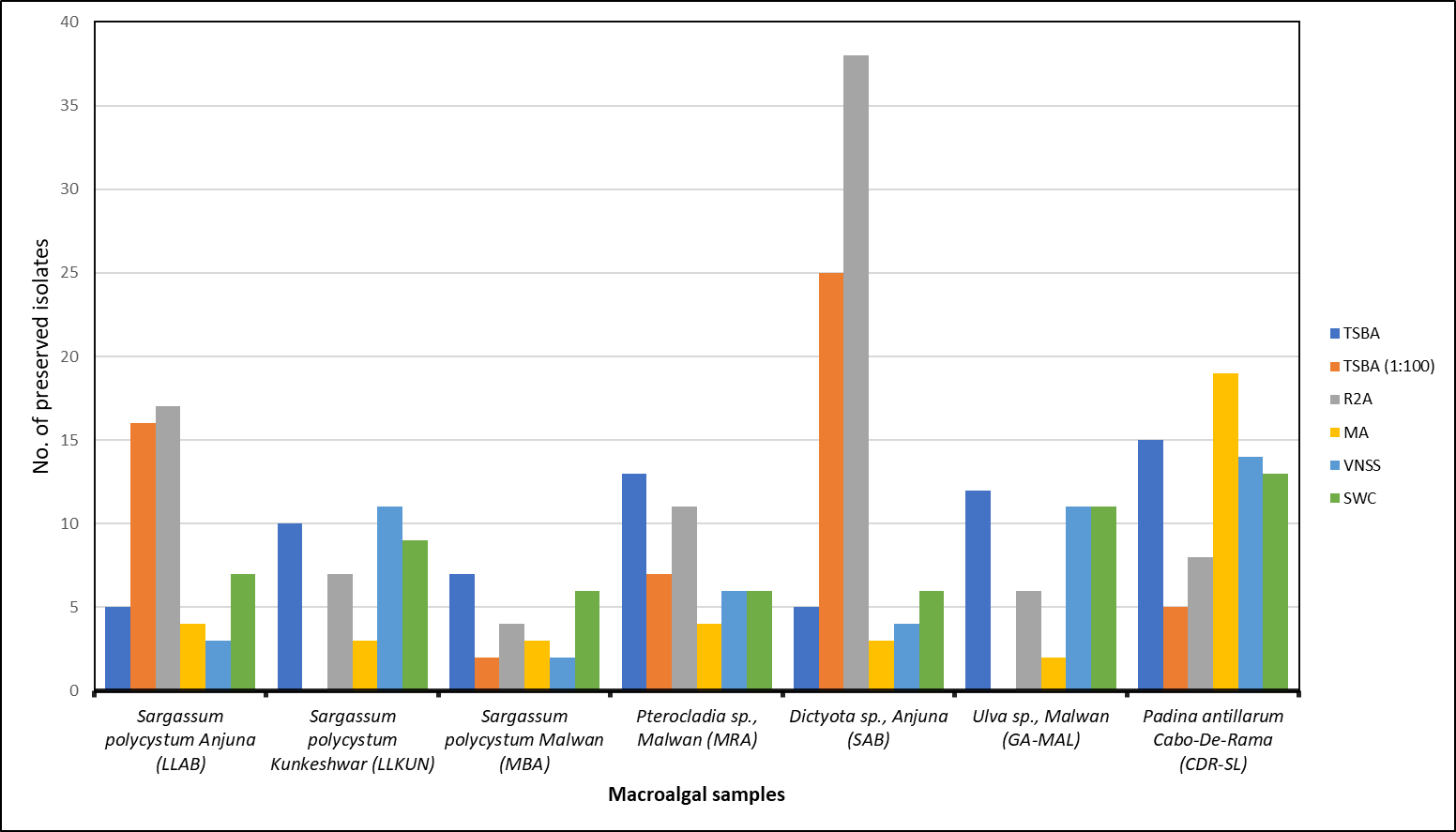

Supplement: Supplementary file 1 [file microorganisms-10-02513-s001.zip › Supplementary figure S1.png]

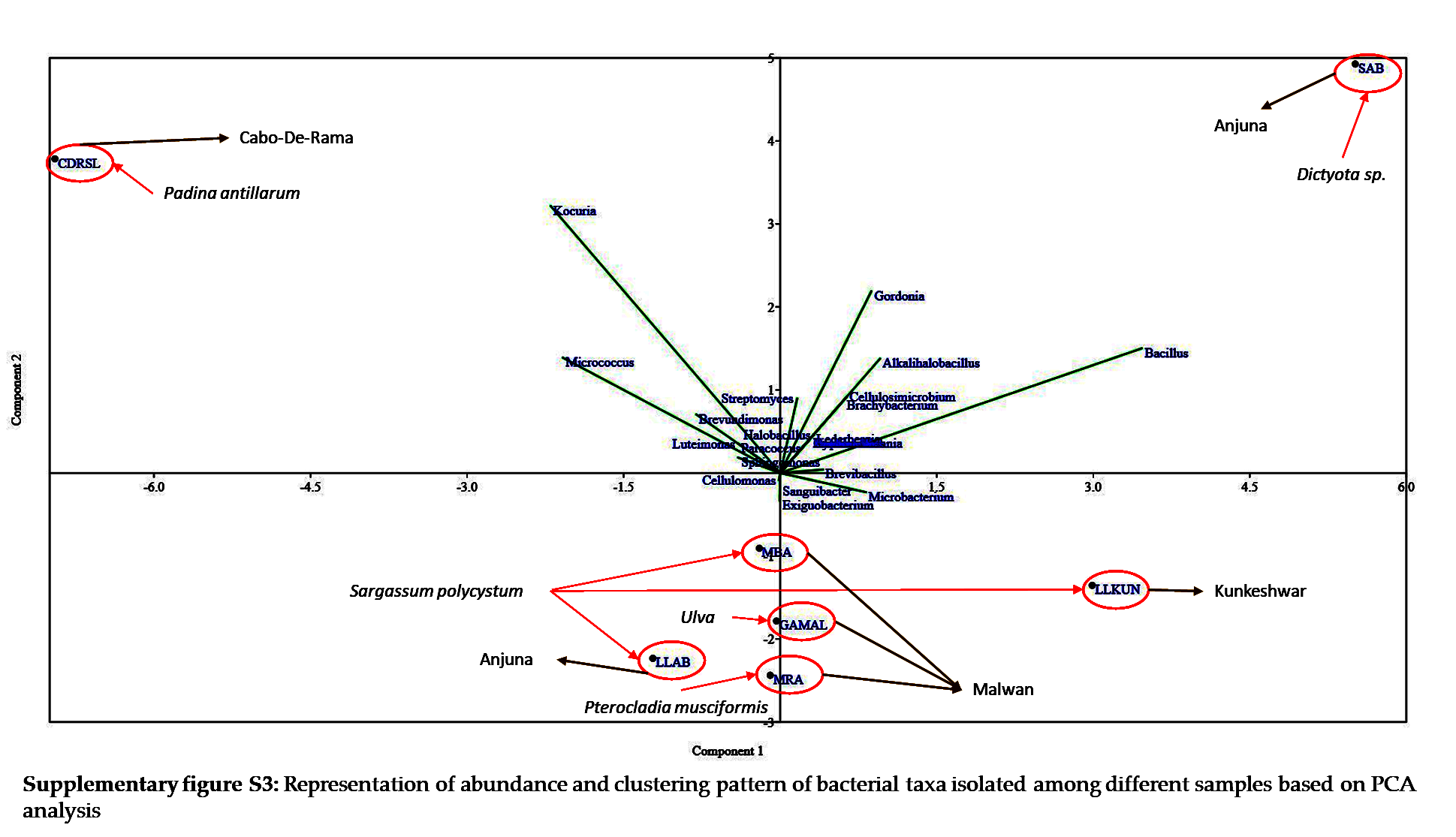

Supplement: Supplementary file 1 [file microorganisms-10-02513-s001.zip › Supplementary figure S3.png]

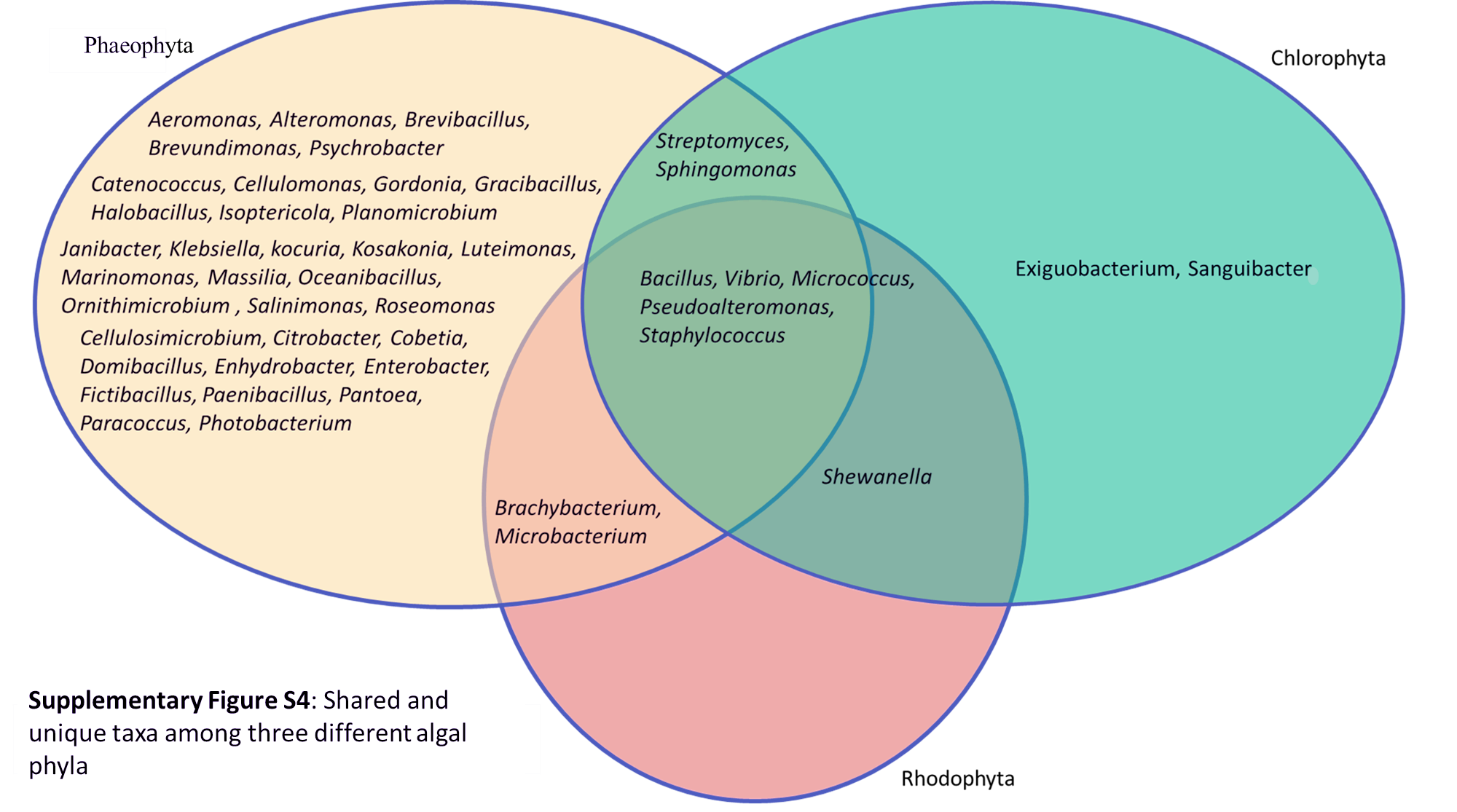

Supplement: Supplementary file 1 [file microorganisms-10-02513-s001.zip › Supplementary figure S4.png]

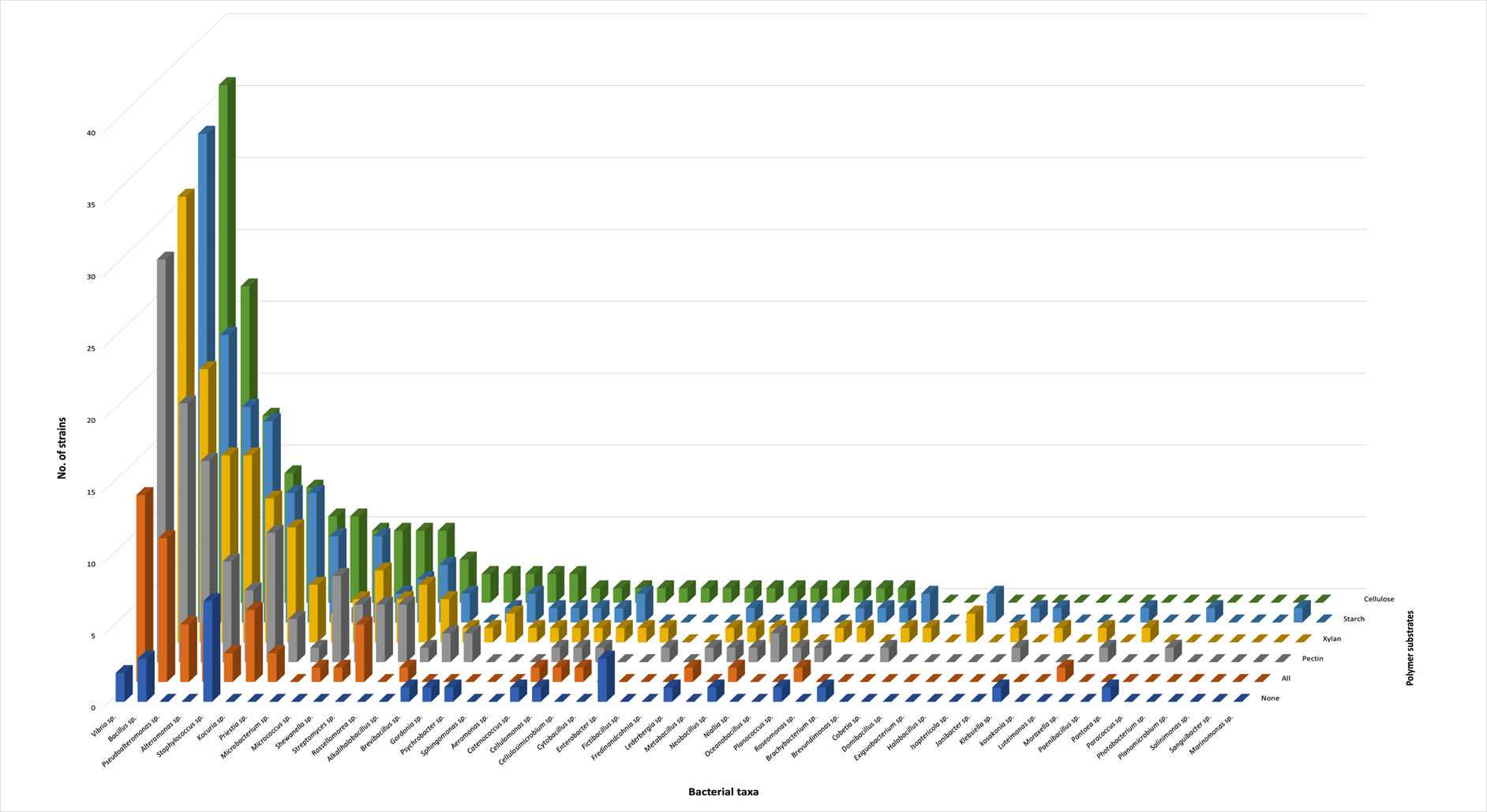

Supplement: Supplementary file 1 [file microorganisms-10-02513-s001.zip › Supplementary figure S5.png]

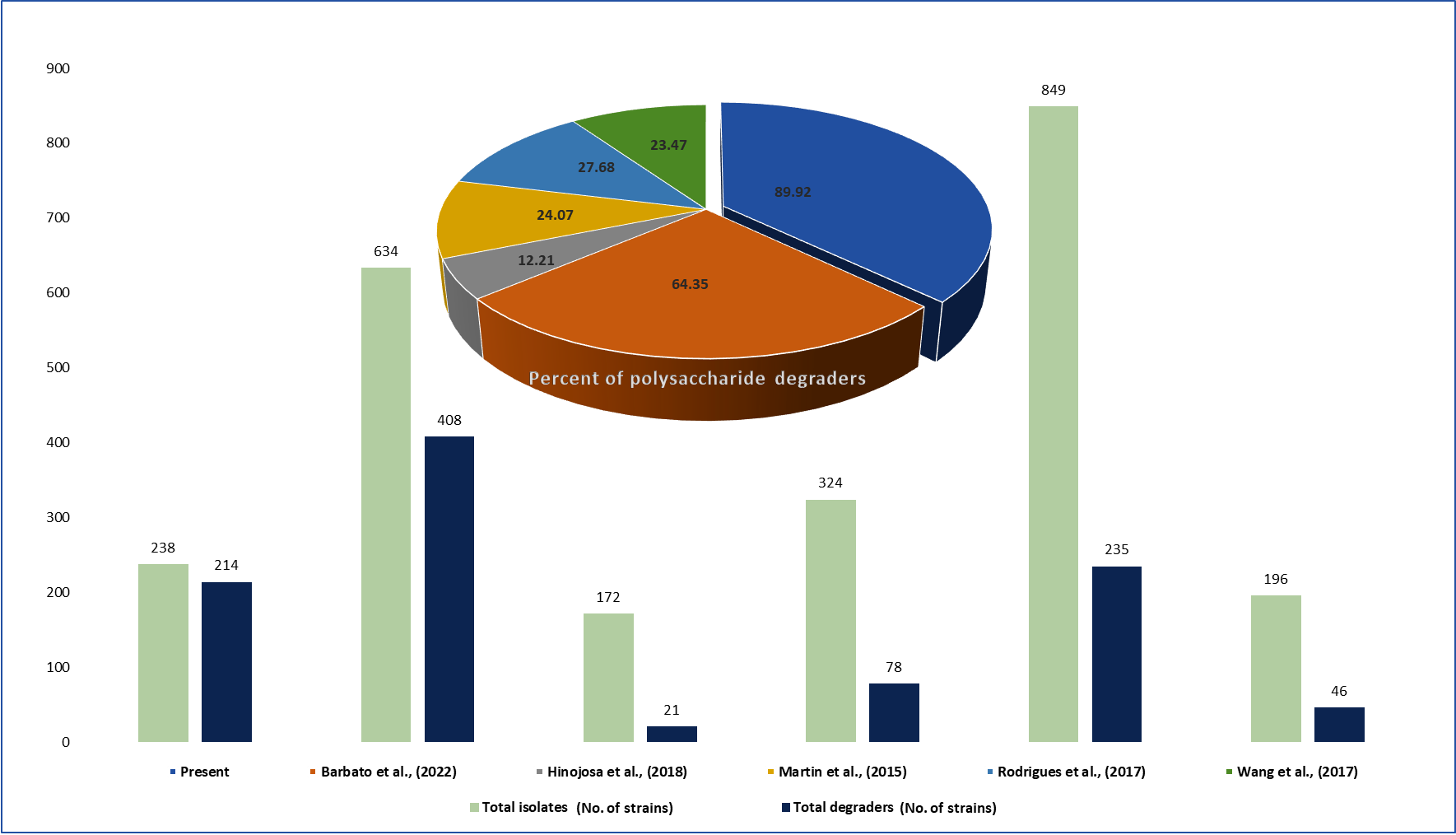

Supplement: Supplementary file 1 [file microorganisms-10-02513-s001.zip › Supplementary figure S6.png]

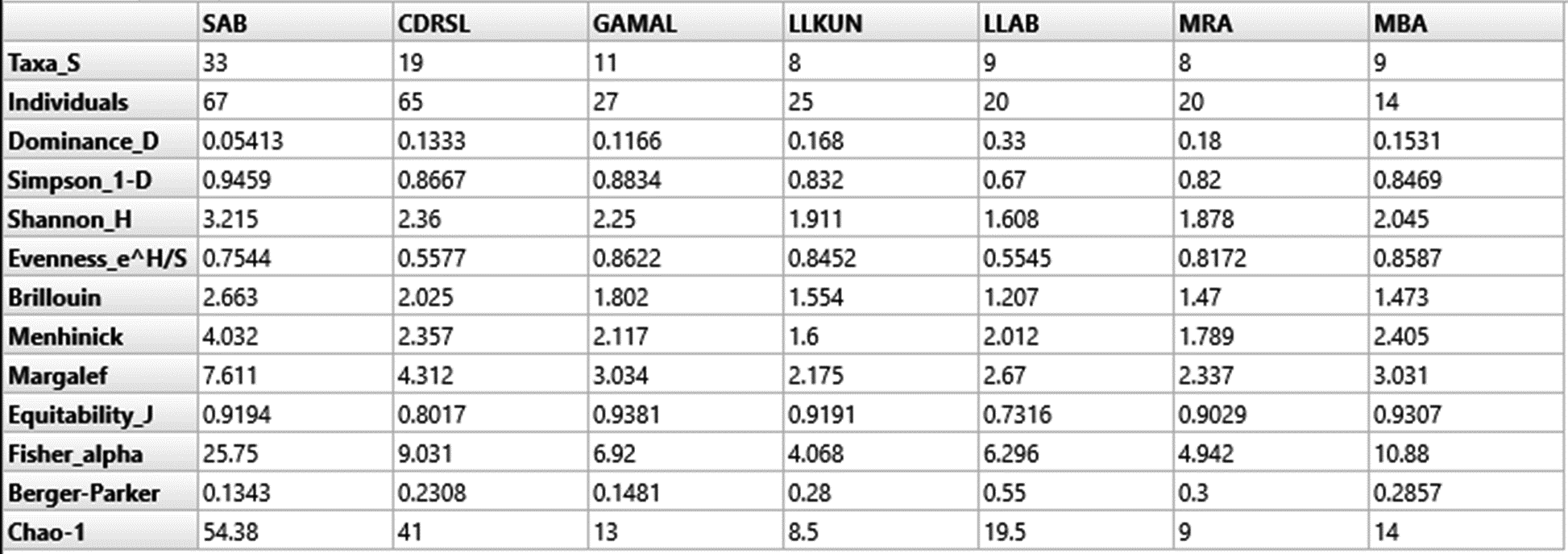

Supplement: Supplementary file 1 [file microorganisms-10-02513-s001.zip › Supplementary figure S7.png]
